# Navigated Dorsal Root Ganglion Stimulation (DRGS) for the Treatment of Chronic Refractory Coccygodynia: A Case Report

**DOI:** 10.7759/cureus.41663

**Published:** 2023-07-10

**Authors:** Natally Santiago, Bernardo A Monaco, Guilherme Santos Piedade, Jonathan Jagid, Joacir G Cordeiro

**Affiliations:** 1 Neurological Surgery, Beneficência Portuguesa de São Paulo, São Paulo, BRA; 2 Neurological Surgery, University of Miami, Miami, USA; 3 Neurological Surgery, CDF - Clinica de Dor e Funcional, São Paulo, BRA; 4 Neurological Surgery, University of Sao Paulo, São Paulo, BRA; 5 Neurological Surgery, Heinrich-Heine-Universität Düsseldorf, Düsseldorf, DEU

**Keywords:** coccygodynia, sacral stimulation, sacral navigation, spine navigation, neuromodulation, dorsal root ganglion

## Abstract

Sacral stimulation is a well-established therapy for urologic neuromodulation. After the advent of dorsal root ganglion (DRG) stimulation, pain surgeons have started to reach this target mostly for pelvic and sacral pain. For those without good surgical experience, sacral foramen puncture, especially S3 and S4, can be a challenge, due to its entry angle and limited C-arm image resolution. In this report, we describe a new technique to utilize sacral navigation using the O-arm approach to guide DRG stimulation implants. We discuss a case of a 53-year-old male patient with refractory coccygodynia, who underwent sacral DRG implantation using neuronavigation. Punctures could be done without the need for multiple attempts to reach the foramen in this patient.

## Introduction

Urogenital and rectal chronic non-malignant pain is usually challenging and sometimes hard to treat and can be very debilitating to patients [1]. Coccygodynia refers to the pain localized to the coccyx and the surrounding structures. It may affect individuals of both genders and all ages but it most commonly affects middle-aged women [2]. Conservative treatment is the first option to manage this condition, and it is successful in most cases [3]. Recent case reports support the use of various neuromodulation techniques to treat refractory coccygodynia [4]. In this report, we describe a new surgical technique to perform dorsal root ganglion (DRG) lead implantation using neuronavigation.

## Case presentation

A 53-year-old male patient presented to our center complaining of refractory coccygodynia. Conservative treatment had failed to relieve his pain. His pain symptoms had begun two years prior to the consultation and no history of trauma was reported. The patient stated that the pain had progressed over the past year. He had been previously evaluated and followed up by pain management physicians and treated with oral medication, transcutaneous electric nerve stimulation (TENS), and two caudal epidural corticosteroid injection (CSI) trials but had resulted in little to no relief. The CSI was performed guided by fluoroscopy and the medication was delivered at the dorsal coccygeal aspect. He described the pain as localized to the “tailbone” and had a visual analog scale (VAS) score of 8-9/10 in severity at the time of presentation. There was no irradiation. Prolonged sitting and transition from sitting to standing made the pain worse. Besides the pain, the patient also endorsed increased urinary frequency, although he denied any urinary or bowel incontinence, lower extremity weakness, or saddle anesthesia. MRI showed no bony deformity or fracture. He was maintained on a pain medication regimen comprising gabapentin 300 mg TID, acetaminophen 325 mg TID, and diclofenac 75 mg BID PRN for pain relief.

The patient presented to our outpatient neurosurgery clinic seeking treatment options for pain relief. After evaluation, the sacral DRG trial was considered. For this purpose, a psychological evaluation was performed to rule out non-organic components or other contraindications to the procedure. The plan involved bilateral S3 and S4 DRG leads placement with tomography-based neuronavigation assistance using permanent leads connected to extensions. If the trial was successful, it would be followed by another procedure to remove the extensions and place the implantable pulse generator (IPG). DRG stimulation trial was performed after receiving informed consent. During the five-day trial, the patient stated that he felt relief in his pain since the stimulation started. Tonic stimulation was delivered at a frequency of 20 Hz, pulse width of 300 us, and amplitude ranging from 0.475 to 1.25 mA. He showed a remarkable pain improvement of more than 50%. Medication as a confounding factor was excluded as they had been held before the procedure. Based on this result, we decided to perform the second stage with the implant of a non-rechargeable IPG (Proclaim DRG, Abbott Laboratories, Chicago, IL) on the left flank. The patient showed remarkable pain reduction from a VAS score of 8-9/10 to 2/10, which was sustained at the six-month follow-up. His quality of life seemed to have improved as the patient reported having a better social life and a full return to work after the stimulation.

Surgical technique

**Figure 1:**
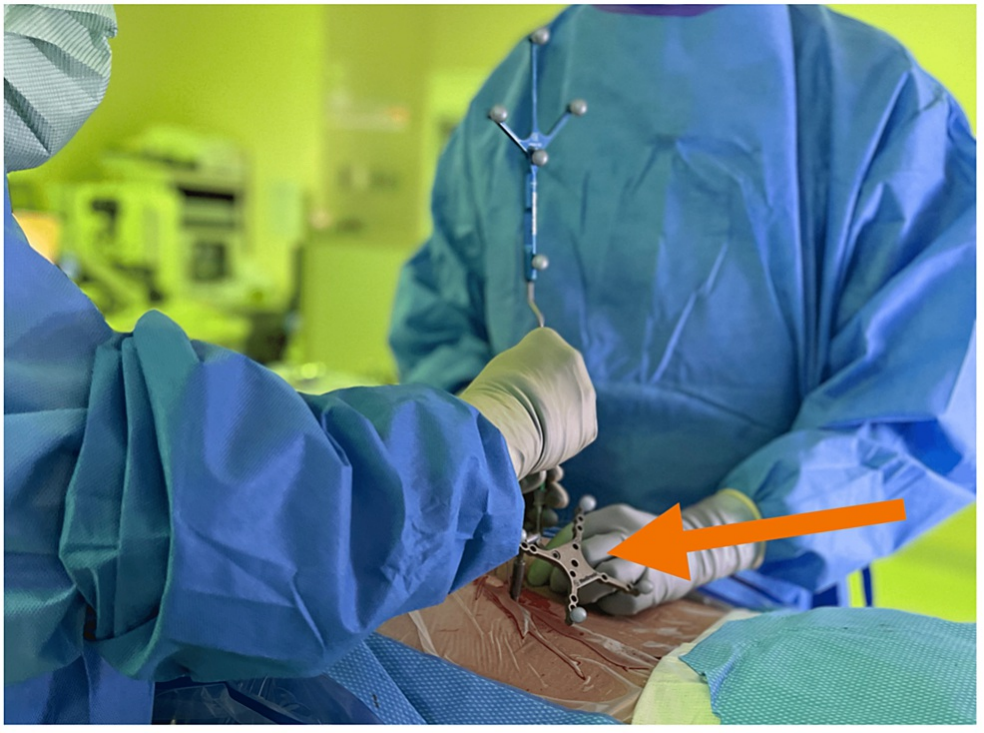
The neuronavigation reference tool is attached to the posterior sacroiliac crest (arrow)

The patient was brought into the operating room and monitored by an anesthesiologist throughout the procedure. Thirty minutes prior to the beginning of the procedure, the patient received ceftriaxone and vancomycin. He was intubated and positioned prone with facial protection on a Jackson table. He was then prepared and draped using chlorhexidine. Local anesthesia was done with lidocaine with epinephrine, and the neuronavigation reference tool was placed on the right posterior sacroiliac crest (Figure 1). 

**Figure 2:**
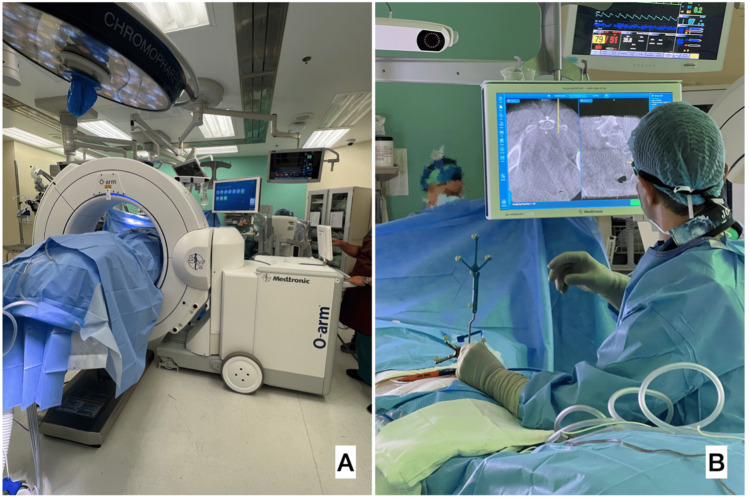
A: O-arm spinning to get image registration. B: Surgeon using the neuronavigational hand tool to determine the best approach to the S3 foramen

Figure 2A shows the intraoperative use of the O-arm. The image was transferred to StealthStation S7 Surgical Navigation System (Medtronic, Minnesota, MN) for intraoperative navigation. The foramina of S3 and S4 were localized with a navigation hand tool (Figure 2B) and insertion of the Tuohy needles was performed using fluoroscopy alternating with neuronavigation.

**Figure 3:**
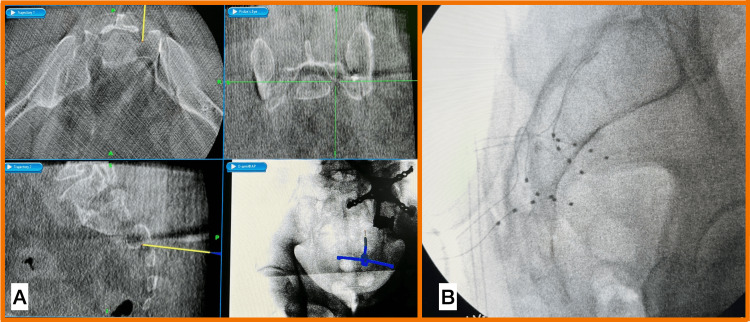
A: Neuronavigation screen is used to determine the best angle to needle insertion into the sacral foramina. B: Fluoroscopic lateral view of the 4-contact DRG leads into S3 and S4 bilaterally DRG: dorsal root ganglion

Needles respected the bone limits preventing them from entering the intrapelvic space. After reaching the epidural space, the curved sheath was advanced under radioscopy fed with the guidewire. Then, the guidewire was swapped with the electrode [4-contact DRG lead (Axium, Abbott Laboratories, Chicago, IL)], which was positioned using fluoroscopy adjacent to the DRG. Then, the electrode stylet was partially retracted, the needle and sheath were rotated, and the electrode was further introduced to create strain reliefs. The same procedure was performed to place the leads in all foramens, for S3 and S4 bilaterally (Figure 3).

Strain relief could not be done despite several attempts in two foramina (right S3 and left S4) given the anatomic features of the sacral canal. After that, the electrodes were tunneled to a left side pocket created on the superior quadrant of the left buttock. They were individually anchored and coupled and tunneled to a more cranial and midline exit around 10 cm distant from the pocket. Impedances were retested after full closure and found to be normal. Once the patient was fully awake, the stimulation was turned on and the patient tolerated it well and was comfortable with it. 

After the successful trial, the patient underwent the second step of the procedure, the IPG implantation. The extensions were discarded, and the leads were connected directly to the IPG. Only four leads were used for the definitive implantation, based on the trial response. Left S4 was removed, and the remaining IPG port was occluded with a sleeve. The impedance test was normal. The neuronavigation system was used only for the lead’s insertion, during the first step of the procedure.

## Discussion

Coccygodynia refers to pain related to pathologies at the most distal segment of the spine, also called the coccyx or tailbone. It may be caused due to various factors, which can be traumatic or non-traumatic, and may even have idiopathic etiologies. However, traumatic coccygodynia is the most common variant [2,5]. The causes of coccyx pain include fracture, dislocation, pelvic floor dysfunction, inflammation (e.g., arthritis), sympathetic nervous system pain, cancer, as well as psychological disorders in some cases. Coccygodynia may affect individuals of both genders and all ages but it most commonly affects middle-aged women. Obesity and female gender are risk factors for developing coccygodynia [2]. Although women are more predisposed to developing this pain, men can also suffer from it. The pain may be burning or gnawing in nature. It is usually worse with prolonged sitting, and when leaning back while seated, and prolonged standing; sometimes, rising from a seated position can initiate the pain. Sometimes, pain can also get worse or be relieved with defecation. Imaging modalities such as X-ray, CT, and MRI can be used for evaluating the presence of traumatic, degenerative, or neoplastic compromise.

The first treatment option usually involves conservative management; it consists of using cushions, application of heat and cold, and non-steroidal anti-inflammatory drugs. TENS can be beneficial in these patients [3]. Minimally invasive treatments such as injections of local anesthetic with CSI around the coccyx can be both diagnostic and therapeutic. Injections are usually given into the sacrococcygeal junction or around the sacrococcygeal ligaments. The ganglion impar block can be used in refractory cases as well as in those associated with malignant neoplasm pain [6]. Surgical amputation of the coccyx just proximal to the sacrococcygeal junction is associated with a high complication rate and failure to relieve the pain. Recent case reports support the use of various neuromodulation techniques to treat refractory coccygodynia, such as conventional spinal cord stimulation and high-frequency spinal cord stimulation.

The successful application of DRG stimulation for coccygodynia has been reported by Giordano et al. [4]. DRG is a key structure in sensory transduction, modulation, and pain transmission, and evidence suggests that it is part of both nociceptive and neuropathic pain states [7,8]. It is a bilateral structure found in every vertebral level and with easy access in the vertebral column. The pseudo-unipolar cells of the DRG were not previously known to be involved in the generation of neuropathic pain. However, in 1983, Wall and Devor demonstrated that electrical impulses originate from within DRG and concluded that it could contribute to producing pain in peripheral nerve damage [9]. DRG can suffer plastic changes when injured, even changing its function and becoming a site of pain signals to the brain [10].

It is important to understand pelvic innervation when considering treatment for coccygodynia and pelvic pain. We have parasympathetic afferents cell bodies in the S2-S4 dorsal root ganglia and they also course within the pelvic splanchnic nerve. The somatic innervation to the pelvis (afferent and efferent innervation) involves the sacral nerve roots (S2-S4) and their ramifications. The posterior perineal musculature is innervated predominantly by nerves from S4 sacral level and also by branches of S4-S5 nerve roots. These branches distribute ramifications to perineal, perianal, and labial or scrotal skin, forming the coccygeal plexus [1]. DRG stimulation seems to be a good alternative for the treatment of pain in more limited areas (e.g., groin) as well as those that are hard or inaccessible to treat with spinal cord stimulation (SCS) (e.g., feet) [11-12]. It is already a well-established therapy and recent developments have expanded the indications for surgery [13-18].

The lead implantation is a percutaneous procedure to access the epidural space and guide into the neuroforamen to reach the DRG. After implantation, the lead can be stabilized by bone structures and ligaments, which makes migration less common [7]. However, reports have shown a 3% lead migration rate. Considering that reaching S3 and S4 sacral foramina may be challenging, especially for less experienced surgeons, neuronavigation may even help reduce surgical time. Another important aspect to note is that lower sacral lead placement is not as common as in other levels. Therefore, except for a few large-volume centers, most of the providers will not have significant experience with this approach and anatomy. 

The neuronavigation system is a useful tool for intraoperative guidance and when planning for a precise surgical approach. Efficient techniques that provide more accuracy and help avoid misplacement of the leads are the goal when trying to improve the results. It is already routinely used in brain and spine surgery with safe definition and helps protection of neurovascular structures, making this alternative a reproducible tactic for surgeons worldwide. In the percutaneous surgical treatment of cranial pain, the trend of using navigation has also been observed. Recent studies have described foramen ovale puncture being done with robotic navigation in balloon compression for trigeminal neuralgia [18,19].

Additionally, neuronavigation can reduce lead misplacement and the necessity for revision. The combined use of the C-arm and O-arm has been frequently employed in spinal instrumentation surgeries, with satisfactory results despite different reports from different centers [20]. It is important to consider that the sample size and the surgeon's experience can significantly influence the results. However, it has been observed that the use of the O-arm provides greater accuracy when compared to the results of instrumentation performed only with the C-arm. It is important to consider that the image obtained by fluoroscopy using the C-arm is two-dimensional, while the O-arm images are three-dimensional, without the need to reposition the patient, which can increase the surgical time to adapt the images. In the case of sacral electrode implants, this makes the use of the O-arm even more preferable for performing the procedure in more challenging anatomical locations such as the S3 and S4 foramina. Costs and availability are still limiting factors for the use of this technology. We presented a case of successful implantation in difficult-to-access foramina with the help of imaging technology, which can be an alternative method in complex implant cases, such as those involving patients with anatomical alterations or spine deformities that limit visualization in routine imaging devices. Costs can vary depending on the institution where the procedure is performed. For instance, non-private institutions that have neuronavigation systems available would be less impacted by their use when compared to private ones.

## Conclusions

Refractory coccygodynia can be very debilitating. Neuromodulation techniques are feasible and safe methods for management in these cases. DRG is part of both nociceptive and neuropathic pain states and DRG stimulation seems to be a good option for the treatment of pain in difficult areas such as the coccyx. Neuronavigation can be a useful tool to guide sacral DRG lead implantation and can provide three-dimensional images that increase accuracy and safety. It is already used in brain and spine surgery and may be especially useful for lead placement in challenging anatomical spaces for surgeons without good clinical experience.
